# Simulation methods with extended stability for stiff biochemical Kinetics

**DOI:** 10.1186/1752-0509-4-110

**Published:** 2010-08-11

**Authors:** Pau Rué, Jordi Villà-Freixa, Kevin Burrage

**Affiliations:** 1 Computational Biochemistry and Biophysics Group, Research Unit on Biomedical Informatics, IMIM/Universitat Pompeu Fabra, c/Dr. Aiguader 88, 08003, Barcelona, Catalonia, Spain; 2 Departament de Física i Enginyeria Nuclear, Universitat Politècnica de Catalunya, Edifici GAIA, Rambla de Sant Nebridi s/n 08222, Terrassa, Barcelona, Spain; 3 Institute for Molecular Bioscience, The University of Queensland, Brisbane, QLD, 4072, Australia; 4 COMLAB and OCISB, University of Oxford, Oxford OX1 3QD, UK

## Abstract

**Background:**

With increasing computer power, simulating the dynamics of complex systems in chemistry and biology is becoming increasingly routine. The modelling of individual reactions in (bio)chemical systems involves a large number of random events that can be simulated by the stochastic simulation algorithm (SSA). The key quantity is the step size, or waiting time, *τ*, whose value inversely depends on the size of the propensities of the different channel reactions and which needs to be re-evaluated after every firing event. Such a discrete event simulation may be extremely expensive, in particular for stiff systems where *τ *can be very short due to the fast kinetics of some of the channel reactions. Several alternative methods have been put forward to increase the integration step size. The so-called *τ*-leap approach takes a larger step size by allowing all the reactions to fire, from a Poisson or Binomial distribution, within that step. Although the expected value for the different species in the reactive system is maintained with respect to more precise methods, the variance at steady state can suffer from large errors as *τ *grows.

**Results:**

In this paper we extend Poisson *τ*-leap methods to a general class of Runge-Kutta (RK) *τ*-leap methods. We show that with the proper selection of the coefficients, the variance of the extended *τ*-leap can be well-behaved, leading to significantly larger step sizes.

**Conclusions:**

The benefit of adapting the extended method to the use of RK frameworks is clear in terms of speed of calculation, as the number of evaluations of the Poisson distribution is still one set per time step, as in the original *τ*-leap method. The approach paves the way to explore new multiscale methods to simulate (bio)chemical systems.

## Background

It is by now very well known that the biochemical kinetics involving small numbers of molecules can be very different to kinetics described by the law of mass action and differential equations [1-3]. This effect is a property of the intrinsic noise of the system and is associated with the uncertainty of knowing when a reaction occurs and what that reaction is. At the molecular level such intrinsic uncertainty is, in turn, a consequence of the stochastic nature of the fluctuations of the potential energy surface for any chemical reaction in the condensed phase [4]. When considering a collection of molecules, the intrinsic noise is accentuated when some chemical species have small numbers, as is often the case in genetic regulatory models where there are small numbers of key transcription factors that can bind to a limited number of operator regions on DNA [5-15]. Kurtz [16] and Gillespie [17] realised this fact and developed discrete methods to deal with this situation. The stochastic simulation algorithm (SSA, see [18] for a review) describes the time evolution of the dynamics of the species in a well-stirred chemically reacting system as a discrete nonlinear Markov process, resulting in an exact method to sample from the probability density function described by the chemical master equation (CME). Gibson and Bruck proposed a more efficient implementation of the SSA called the next reaction method [19].

The basic idea of the SSA is that at each time point a waiting time to the next reaction and the most likely reaction to occur must be sampled from a joint probability density function leading to an appropriate update of the state vector. But if the rate constants and/or the numbers of molecules in the system are large then the waiting time (time step, *τ*) can be very small [18]. Because of this Gillespie [20] introduced the Poisson *τ*-leap method, in which all reactions are allowed to fire in a given *τ *with a frequency extracted from a Poisson distribution. Since then many extensions of this idea have been developed. Cao et al. [21] have considered efficient mechanisms for selecting *τ *and have developed implicit methods suitable for simulating stiff systems. Tian and Burrage [22] introduced a modification of Poisson *τ*-leap methods known as Binomial *τ*-leap methods that avoids the issue of obtaining negative molecular numbers from which Poisson *τ*-leap methods can suffer. Chatterjee et al. [23] and Auger et al. [24] have considered modifications to Binomial *τ*-leap methods that improve some of the implementation aspects. On the other hand, Monk [25] and Mackey [26] noted the importance of representing delays, especially when representing processes such as transcription and translation. Accordingly, Bratsun et al. [12] and Barrio et al. [27] developed a delayed version of the Stochastic Simulation Algorithm. Leier et al. [28] and Anderson [29] extended these ideas to a *τ*-leap setting.

Although *τ*-leap methods can, in some cases, substantially improve computational efficiency compared with the SSA, when there is moderate stiffness in the system the efficiencies can be quite poor. One could resort to implicit *τ*-leap methods but then there are considerable implementation issues and subtleties. A different approach is to explore ideas from the numerical ODE (ordinary differential equations) and numerical SDE (stochastic differential equations) communities. Thus, with ODEs it is well known that stiffness leads to a step size restriction when using explicit methods and many classes of efficient implicit methods have been designed [30]. However, in the case of moderately stiff systems explicit Runge-Kutta methods with extended stability regions along the negative real axis have proven to be especially effective [31,32]. Runge-Kutta methods are a class of one step methods which gain their efficacy by computing intermediate approximations to the solution within a step. Explicit Runge-Kutta methods with extended stability regions are based on explicit Runge-Kutta methods whose stability function is a shifted and scaled Chebyshev polynomial or some variant thereof. In the stochastic setting, there are some subtleties designing fully implicit methods due to possible unboundedness of the solution as the Wiener increment can take positive or negative values with equal likelihood [33]. Thus most methods are semi-implicit, that is implicit in the deterministic component. Abdulle and Cirilli [32] have, with some success, extended the ideas of explicit Chebyshev methods with extended stability regions to the SDE setting via their class of S-ROCK methods.

Here, we use the Runge-Kutta formulation to construct methods with large stability regions so that efficiencies are gained by allowing larger stepsizes. We note that this is exactly what Abdulle and Cirilli [32] do in the SDE setting, that is they use a Runge-Kutta formulation to construct methods with excellent stability properties and even though these methods are only weak order 1 they perform very well. It is noteworthy that in this work we are not using the Runge-Kutta formulation to get second order accuracy for *τ*-leap methods. This seems to be a difficult problem, just as it is the case for SDEs and will probably require double integrals of compensated processes to be simulated. In fact, Abdulle and Cirilli [32] also note that it is very difficult to construct weak order 2 methods with good stability properties and to our knowledge at the moment no such methods exist in the SDE setting. Note that in a stochastic setting we judge order of accuracy through two mechanisms: strong order (where trajectories are compared with the true solutions) and weak order (where moments are compared). Often a numerical method may have a higher weak order than its strong order. The Euler-Maruyama method is a case in point with weak order one and strong order a half.

Thus, in this paper, we explore a series of fully explicit multistage Runge-Kutta methods with extended stability for a fixed *τ*-leap stochastic simulation schema. Our methods involve the same number of Poisson evaluations per integration step as in the original *τ*-leap formulation but allow increasingly larger step sizes at the cost of an increasing series of deterministic evaluations in the internal stages. First we give some background on Runge-Kutta methods for ODEs and SDEs. In section Results we extend these ideas to the *τ*-leap methods and present a stability analysis for linear chemical kinetics, including its practical implementation. In section Numerical results we present numerical results for both the linear case and the classical stiff system described by the Schlögl reaction [34]. Finally, in section Discussion we discuss further implications of this work and, in particular, possible extensions to multiscale modelling.

### Review of Runge-Kutta methods for SDEs and ODEs

#### Stability region for RK methods applied to ODEs

Consider the system of initial value ODEs given by

(1) $$y^{\prime }(t)=f(t,y),\;\;y(t_{0})=y_{0}.$$

The class of *s*-stage Runge-Kutta (RK) methods for approximating the solution to (1) is given by

(2) $$\begin{array}{c} Y_{i}=y_{n}+h\sum_{j=1}^{s}\alpha_{ij}f(t_{n}+\omega_{j}h,Y_{j}),\text{for }i=1,\ldots ,s,\\ y_{n+1}=y_{n}+h\sum_{j=1}^{s}\beta_{j}f(t_{n}+\omega_{j}h,Y_{j}), \end{array}$$

where *h *is the time step. This class of methods is characterised by the Butcher tableau

$$\begin{array}{cc} w & A\\ & b^{T} \end{array}$$

where **b**^T ^= (*β*_1_,...,*β*_*s*_), **w **= **Ae **and **e **= (1,...,1)^T^. Here **A **is the matrix with entries *α*_*ij *_and **w **is the column vector **w**^T ^= (*w*_1_,...,*w*_s_)^T^. A Runge-Kutta method is said to be explicit if the *s *× *s *matrix **A **is strictly lower triangular. The method parameters are usually chosen so that a Runge-Kutta method has appropriate efficiency, order and stability characteristics. The **Y**_*i *_are considered to be approximations to the solution at the intermediate points *t*_*n *_+ *w*_*i*_*h *for *i *= 1,...*s*.

In a stability setting an RK method is often applied to the linear, scalar test equation

(3) y′=λy, Re[λ]≤0.

In which case it is easily seen that (2) gives rise to

$$y_{n+1}=R(h\lambda )y_{n},$$

Where

(4) $$R(z)=1+zb^{\text{T}}{\left(I-\;Az\right)}^{-1}e\text{.}$$.

Here *R*(*z*) is the so-called *stability function*. This function can be extended to a linear *N*-dimensional equation **y' **= **Λy **in which case it becomes a matrix function of the *N *× *N *matrix **Λ**:

(5) $$\begin{array}{l} R(h\Lambda )=I_{N}+(hb^{\text{T}}⊗\\ \;\;\Lambda {\left(I_{s}⊗I_{N}-hA⊗\Lambda \right)}^{-1}(e⊗I_{N})), \end{array}$$

where **e **is the unit vector, **I**_***s ***_is the identity matrix of order *s *and ⊗ represents the Kronecker tensor product such that the (*i, j*) element of **A **⊗ **B **is *a*_*ij*_**B**. Notice that, if **Λ **is a scalar value and taking *z *= *h***Λ**, *R*(*z*) would be a scalar and take the form (4). Therefore we can refer to *R *seamlessly irrespective of whether the argument is a matrix or a scalar.

In the case of an explicit method, as **A **is a strictly lower triangular *s *× *s *matrix, its *s*th power is **A**^***s ***^= **0**. Therefore, equation (4) can be expanded into a finite power series for **A**:

(6) $$\begin{array}{c} R(z)=1+\sum_{j=1}^{s}z^{j}b^{\text{T}}A^{j-1}e\\ =1+\sum_{j=1}^{s}r_{j}z^{j}, \end{array}$$

where *r*_*j *_= **b**^T^**A**^*j*-1^**e**, *j *= 1,...,*s*. Hence, *R*(*z*) is a polynomial of at most degree *s *for any explicit method.

Since (3) is asymptotically stable for all Re [*λ*] < 0, the stability region of a Runge-Kutta method is defined as

(7) S={z=hλ∈ℂ:|R(z)| ≤1}.

#### Stability region for RK methods applied to SDEs

In the case of stochastic differential equations (SDEs), we consider the general *m *dimensional form

(8) $$\begin{array}{l} \text{d}y\text{(}t\text{) = }f\text{(}t\text{, }y\text{)d}t\text{+ }g\text{(}t\text{, }y\text{)d}W\text{(}t\text{),}\\ y\text{(}t_{\text{0}}\text{) = }y_{\text{0}}, \end{array}$$

where **W**(*t*) = (*W*_1_(*t*),...,*W*_*d*_(*t*))^T ^is a vector of *d *independent Wiener processes in which an individual Wiener process has the properties

$$\begin{array}{c} E\left[W(t)\right]=0,\;\forall t,\\ \text{Var}\left[W(t)-W(s)\right]=t-s,\;t>s \end{array}$$

and non-overlapping Wiener increments are independent of one another. A sample of a Wiener increment *W*(*t *+ *h*) - *W*(*t*) is simulated from a Normal random variable with mean 0 and variance h, *N*(0, *h*).

Equation (8) can arise as the limit of a discrete process through the concept of a diffusion process in which case **f **(*t*, **y**) will represent the mean of this process and **g**(*t*, **y**) is the *m *× *d *matrix such that **gg**^T ^is the covariance. Equation (8) can be interpreted in several ways (see [35] for an introduction to SDEs), depending on which integral definition is used. Two such interpretations lead to Itô and Stratonovich forms of SDEs. In the Itô setting an integral is approximated by summing, over a partition, the areas of a rectangle with width the increment of the Wiener process on that subinterval and height the value of the integrand at the lefthand point of each subinterval whereas in the Stratonovich setting the integrand is evaluated at the midpoint of each interval. If (8) is interpreted in the Itô sense then the simplest numerical algorithm is given by

(9) $$y_{n+1}=y_{n}+hf(t_{n},y_{n})+\Delta W_{n}g(t_{n},y_{n}),$$

where Δ**W**_*n *_= (Δ*W*_1_,....Δ*W*_*d*_)^T ^and Δ*W*_*i  *_:= *W*_*i*_(*t*_*n *_+ *h*) - *W*_*i*_(*t*_*n *_), *i *= 1,...,*d *are normally distributed random numbers with mean 0 and variance *h*. This method is known as the Euler-Maruyama method and it is known to have strong order (pathwise order) $\frac{1}{2}$ and weak order (moment order) 1.

As with the deterministic case, the quality of a numerical method can be partly characterised by its stability region associated with the scalar, linear test equation

(10) $$\text{d}y=ay\text{d}t+by\text{d}W,\;y(0)=y_{0}.$$

The solutions of (10) in the Itô and Stratonovich cases are, respectively,

$$\begin{array}{l} y_{I}(t)=e^{\left(a-\frac{1}{2}b^{2}\right)t+bW(t)}y_{0}\;\text{and}\\ y_{s}(t)=e^{at+bW(t)}y_{0}. \end{array}$$

In the later case, the solution is mean square stable $\left(\lim_{t\to \infty }E\left[|y_{S}(t){|}^{2}\right]=0\right)$ if Re [*a*] + Re [*b*^2^] ≤ 0.

A very general class of stochastic Runge-Kutta methods [36] was constructed for the solution of (8) which, when applied to the scalar test SDE (10) produces

$$E\left[|y_{n+1}{|}^{2}\right]=R(p,q)\;E\left[|y_{n}{|}^{2}\right],$$

where *R *is a multinomial in *p *and *q *if the method is explicit and where *p *= *ha*, $q=\sqrt{hb}$. Analogous to the deterministic case, the mean square stability region of a method is defined as

S={p,q∈ℂ:R(p,q)≤1}.

In the case of the Euler-Maruyama method

$$R(p,q)=|1+p{|}^{2}+|q{|}^{2}$$

and in the (*p, q*) plane, with *p, q *∈ ℝ, the stability region is a circle of radius 1 centered in (-1,0).

## Results

### The *τ*-leap Runge-Kutta framework with bounded variance and extended stability domain

As stated in the Background section, the SSA describes the time evolution of a vector of integer numbers of molecules in the presence of intrinsic noise. More formally, suppose that there are *N *chemical species *S*_1_,...,*S*_N _undergoing *m *chemical reactions. Let *X*_*i*_(*t*), *i *= 1,...,*N *denote the number of molecules of species *S*_*i *_and **X**(*t*) = (*X*_1_(*t*),...,*X*_*N*_(*t*))^T^. Now any set of chemical reactions is uniquely characterised by two sets of quantities. These are the update (stoichiometric) vectors *ν*_1_,...,*ν*_*m *_for each of the *m *reactions and the propensity functions *a*_1_(**X**(*t*)),...,*a*_*m*_(**X**(*t*)), which are proportional to the probabilities of each of the reactions occurring. For example, given the reaction

$$A+B\overset{c}{\to }C$$

then **X**(*t*) = (*A*(*t*), *B*(*t*), *C*(t))^T^, *ν*_1 _= (-1, -1, 1)^T ^and *a*_1_(**X**(*t*)) = *cA*(*t*)*B*(*t*).

Given **X**(*t*) at time *t*, the SSA determines a waiting time *τ *to the next reaction assuming an exponential waiting time distribution $e^{-\tau a_{0}(X(t))}$, where $a_{0}(X(t))=\sum_{j=1}^{m}a_{j}(X(t))$, and then selects the most likely reaction, say *k*, based on the relative sizes of *a*_1_(***X***(*t*)),...,*a*_*m*_(***X***(*t*)). The state vector is then updated as

$$X(t+\tau )=\;X(t)+\;\nu_{k},$$

and the algorithm repeats.

Since a typical stepsize (waiting time) is of the size 1/*a*_0_(**X**(*t*)), this can be very small if some of the rate constants are large and/or some species have large numbers of molecules. Accordingly *τ*-leap methods attempt to take a larger step size in which all the reactions can occur based on a certain frequency. This can be written as

(11) $$X_{n+1}=X_{n}+\sum_{j=1}^{m}\;\nu_{j}K_{j}.$$

Gillespie [20] chose the number of *R*_*j *_reactions per step, *K*_*j *_, as coming from a Poisson distribution with mean *τa*_*j*_(**X**_*n*_), that is

(12) $$K_{j}\sim P(\tau a_{j}(X_{n})).$$

Using the so-called compensated process given by

(13) L(τ,x)=P(τx)−τx,

which satisfies E[*L *(*τ*, *x*)] = 0 and E [*L *(*τ*, *x*)^2^] = *τx*, equation (11) can be restated as

(14) $$X_{n+1}=X_{n}+\tau f(X_{n})+\sum_{j=1}^{m}\nu_{j}L(\tau ,a_{j}(X_{n})),$$

Where $f(x)=\sum_{j=1}^{m}\nu_{j}a_{j}(x)$.

As noted by Gillespie [20] and Tian and Burrage [22], and as a consequence of the Law of Large Numbers, as *xτ *→ ∞, *L*(*τ*, *x*) converges to a normal random variable with zero mean and variance *τx*, *N*(0, *τx*), and this can be considered as a sample $\sqrt{x}\Delta W_{n}$ of $\sqrt{x}N(0\text{,}\tau )$. Substituting this into (14) gives

(15) $$X_{n+1}=X_{n}+\tau f(X_{n})+\sum_{j=1}^{m}\nu_{j}\sqrt{a_{j}(X_{n})}\Delta W_{j}.$$

This is precisely the Euler-Maruyama method applied to the SDE

(16) $$\text{d}X=\sum_{j=1}^{m}\nu_{j}a_{j}(X)\text{d}t+\sum_{j=1}^{m}\nu_{j}\sqrt{a_{j}(X)}\text{d}W_{j}.$$

Thus in the continuous limit the Poisson *τ*-leap method can be viewed as the Euler-Maruyama method applied to a form of the Chemical Langevin Equation. Indeed Li [37] has shown that the Poisson *τ*-leap method has mean square strong order $\frac{1}{2}$ and weak order 1 and this is consistent with the previous remarks. In addition, equation (16) is a particular case of the general SDE

$$\text{d}X=\text{f}(X)\text{d}t+\sum_{k=1}^{m}g_{k}(X)\text{d}W_{k}.$$

These relationships naturally lead to the introduction of the class of Runge-Kutta *τ*-leap methods which bears a relationship, similar to the one discussed above, to the general class of Stochastic Runge-Kutta methods for solving SDEs [36]. This general class of explicit *s*-stage Runge-Kutta *τ*-leap methods takes the form

(17) $$\begin{array}{c} d_{n}=\sum_{j=1}^{m}\nu_{j}L(\tau ,a_{j}(X_{n}))\\ Y_{i}=X_{n}+\tau \sum_{j=1}^{i-1}\alpha_{ij}f(Y_{j})+\omega_{i}d_{n},i=1,\ldots ,s\\ X_{n+1}=X_{n}+\tau \sum_{j=1}^{s}\beta_{j}f(Y_{j})+d_{n} \end{array}$$

where *L*(*τ*, *x*) is given by (13) and $f(x)=\sum_{j=1}^{m}\nu_{j}a_{j}(x)$ represents the drift or expected stepchange. As our focus is explicit methods, the matrix **A **is strictly lower diagonal. We note that (17) requires the same number of samples of Poisson random variables per step as the Poisson *τ*-leap method.

The Poisson *τ*-leap method given by (11) and (12) is equivalent to (17) with

$$\begin{array}{ccc} s=1, & A=0, & \beta_{1}=1. \end{array}$$

Indeed any Runge-Kutta method for solving an ODE can be incorporated into this framework. We also note that other methods proposed in the literature can be put into this framework. For example, the midpoint method of Gillespie [20] can be represented with *s *= 2, **b**^T ^= (0, 1), **w **= (0, 0.5)^T ^and where the row-wise entries of **A **are 0, 0, 0.5, 0.

#### The linear case

As in the case of stability settings in the ODE and SDE regimes, we analyse (17) when applied to linear kinetics, which in this case are described by sets of unimolecular reactions. A general set of *m *unimolecular reactions can be described by *m *propensity functions given by the following linear functions

(18) $$a_{j}(x)=\sum_{i=1}^{N}c_{ij}x_{i}=c_{j}{}^{T}x,\;j=1,\ldots ,m,$$

where **x **is the state vector of dimension *N *and $c_{j}={\left(c_{1_{j}},\ldots ,c_{N_{j}}\right)}^{T}$, *j *= 1,...,*m *are *m *vectors of dimension *N *defining the propensities. A more convenient way to describe this linear kinetics system is by using the *N *× *N *matrix **W**

$$W=\sum_{j=1}^{m}\nu_{j}c_{j}{}^{T},$$

so that now the drift or expected step-change can be represented as

$$f(x)=\sum_{j=1}^{m}\nu_{j}c_{j^{\text{T}}}x=Wx.$$

If the Runge-Kutta method for ODEs underlying a Runge-Kutta *τ*-leap method (17) has stability function given by (4), then when the latter is applied to (18) we show (Additional file 1) that

(19) $$E[X_{n+1}]=R(\tau W)E[X_{n}],$$

where *R *is the multidimensional version of (4) given by (5). Note that this is a natural generalization of the deterministic case when a Runge-Kutta method is applied to the problem **y' **= **Λy **giving **X**_*n *_= *R*(*h***Λ**)**X**_*n*-1_. Thus with fixed stepsize *τ*

(20) $$E[X_{n}]=R{\left(\tau W\right)}^{n}E[X_{0}].$$

Therefore, boundedness in the mean requires that the spectral radius, *ρ*, of *R*(*τ***W**) satisfies

ρ(R(τW))≤1.

In order to analyse the framework (17) from the perspective of both mean and variance behaviour we consider the reversible isomerisation reaction with fixed total number of molecules given by

(21) $$S_{1}\overset{k_{1}}{\underset{k_{2}}{⇄}}S_{2},$$

as the linear scalar test equation. It is easy to see that this system is a analogous to (3) for ODEs and (10) to SDEs with constant nonzero term. The system is chosen to have constant nonzero term in order to compare its variance, which otherwise would fade to zero, to the variance given by the framework methods (17). In this case

(22) $$W=\left(\begin{array}{cc} -k_{1} & k_{2}\\ k_{1} & -k_{2} \end{array}\right).$$.

For this set of reactions, the Chemical Master Equation (which describes the probability density function associated with the evolving Markov process **X**) can be solved analytically [18,38]. In particular, it can be shown that the stationary state $X*={\left(X_{1}^{*},X_{2}^{*}\right)}^{T}$ has a probability density function (PDF) that follows a binomial distribution with

$$P(X_{1}^{*}=x)=\frac{T!}{x!(T-x)!}p^{x}{\left(1-p\right)}^{N-x}$$

Where

$$p=\frac{k_{2}}{k_{1}+k_{2}}$$

and *T *= *X*_1_(*t*) + *X*_2_(*t*) is the (fixed) total number of molecules in the system. Thus from the properties of the binomial distribution with **e **= (1, 1)^T^

(23) $$\begin{array}{c} E\left[X*\right]=\frac{T}{k_{1}+k_{2}}{\left(k_{2},k_{1}\right)}^{\text{T}}\\ \text{Var}\;\left[X*\right]=\frac{T}{{\left(k_{1}+k_{2}\right)}^{2}}k_{1}k_{2}e. \end{array}$$

In the case of non-negative coefficients in the underlying RK method and for constant *τ *one can show (see details in Additional file 1) that if (17) is applied to (21) with constant *τ *such that |*R*(*z*)| < 1, *z *= -*τ*(*k*_1 _+ *k*_2_), then in the limit as n → ∞ the mean vector converges to the theoretical mean, that is

$$\underset{n\to \infty }{\lim}E[X_{n}]=E[X*].$$

Note that with the constraint |*R*(*z*)| < 1, *z *= -*τ*(*k*_1 _+ *k*_2_) then the spectral radius of *R*(*τ***W**) is less than or equal to 1, and as there is only one eigenvalue equal to one hence we have boundedness of the mean.

Furthermore, if Var [**X**_∞_] denotes the variance of the new method at steady state (*X*_1 _and *X*_2 _have the same variance) and if *R*^2^(*z*) ≠ 1, *z *= -*τ*(*k*_1 _+ *k*_2_), then (see details in Additional File 1)

$$\text{Var}[X_{\infty }]=\psi (z)\text{Var}[X*],$$

where

(24) $$\psi (z)=\frac{2}{z}\left(\frac{R(z)-1}{R(z)+1}\right).$$

We call this the relative variance at the stationary state associated to *R*.

Let us consider some particular cases of this result:

**Poisson *τ*-leap **For this method *R*(*z*) = 1 + *z *and $\psi (z)=\frac{1}{1+\frac{1}{2}z}$. Thus, the equilibrium variance doubles at *z *= -1, it rises fourfold at *z *= -1.5 and is unbounded at *z *= -2.

**Two stage methods with ***α*_21 _≠ 0 For the family of explicit two-stage methods with *α*_21 _≠ 0

$$\begin{array}{cc} & \begin{array}{cc} 0 & 0\\ \alpha_{21} & 0 \end{array}\\ & \begin{array}{cc} \beta_{1} & \beta_{2} \end{array} \end{array}$$

the stability function is *R*(*z*) = 1 + *z *+ *γz*^2^, where *γ *= *β*_2_*α*_21 _and the variance behaviour is determined by

$$\psi (z)=\frac{1+\gamma z}{1+\frac{1}{2}z+\frac{\gamma }{2}z^{2}}.$$

In this case we have one free parameter of the method, *γ*, which allows us to control both the stability function *R *and the relative variance at steady state. We might be interested in setting *γ *to a value that both allows large time-steps to be used (by maximising the region (-*l*, 0] for which *z *fulfils |*R*(*z*)| < 1) and keeps the relative variance, *ψ*(*z*) close to one. In the case *γ *≤ $\frac{1}{8}$, *ψ *grows as *z *becomes more negative. More interesting is the case *γ *>$\frac{1}{8}$, where the maximum and minimum of *ψ *occur for $1+\gamma z=\pm \sqrt{2\gamma }$, respectively and in this case

$$\frac{-2\gamma }{\sqrt{8\gamma }+1}\leq \psi (z)\leq \frac{2\gamma }{\sqrt{8\gamma }-1}.$$

Constraining *ψ *to be around 1 with a certain fixed tolerance ϵ, |*ψ*(*z*) -1| < ϵ, for a range *z *∈ (-*l*, 0] to be maximised is achieved with

$$\gamma =(1+\epsilon )\left[\left(\frac{1}{2}+\epsilon \right)-\sqrt{\epsilon (1+\epsilon )}\right]$$

and with a *stability region *(-*l*, 0] with

$$l=-\left[\frac{1}{1-\epsilon }-\frac{1}{2\gamma }-\sqrt{{\left(\frac{1}{2\gamma }+\frac{1}{1-\epsilon }\right)}^{2}-\frac{2}{\gamma }}\right].$$

For instance, for 0.5 <*ψ*(*z*) < 1.5, setting *γ *= 0.20096 gives a maximum stability region of (-3.68026, 0] and thus the method

$$\begin{array}{ccc} & 0 & 0\\ & 0.20096 & 0\\ & 0 & 1 \end{array}.$$

This is the methodology we propose in the following section for the derivation of particular Runge-Kutta methods with *s *steps. Note that if we required the same limitation on the variance with the standard Poisson *τ*-leap method we could only take $z\in (-\frac{2}{3},0]$. Thus with the two stage method we can take a stepsize almost six times as large.

**Implicit midpoint rule **For the implicit midpoint rule

(25) $$\begin{array}{c} \begin{array}{cc} \frac{1}{2} & \frac{1}{2}\\ & 1 \end{array}\\ R(z)=\frac{1+\frac{1}{2}z}{1-\frac{1}{2}z}\;\text{and}\;\psi (z)=1,\forall z. \end{array}$$

This was first shown by Cao *et al*. [38]. In fact only those Runge-Kutta methods that have a stability function given by (25) can preserve the variance exactly for linear problems. These methods include the implicit midpoint and trapezoidal rules and have to be implicit.

#### Methods with bounded variance and extended stability domain

For the general case of *s *stages we require *ψ*(*z*) to be as close to 1 as possible for as large a range of *z *as possible, this is, for as large a range of *z *fulfilling the stability condition |*R*(*z*)| < 1). We proceed by first showing that if we consider a bound on the relative variance, *ψ*, around one, we automatically fulfil the stability conditions for a certain range. In this sense, let ϵ ≥ 0 (and ϵ < 1), we impose the constraint

(26) |ψ(z)−1| <ϵ

and optimise the value of *l*_*s*, ϵ _such that the range for which this holds is (-*l*_*s*, ϵ_, 0].

Noticing from (24) that

(27) $$R(z)=\frac{1+\frac{z}{2}\psi (z)}{1-\frac{z}{2}\psi (z)},$$

inequality (26) can be restated in terms of *R*(*z*)

(28) $$\frac{1+\frac{z}{2}(1+\epsilon )}{1-\frac{z}{2}(1+\epsilon )}<R(z)<\frac{1+\frac{z}{2}(1-\epsilon )}{1-\frac{z}{2}(1-\epsilon )},$$

with *z *∈ (-*l*_*s *ϵ_, 0]. Hence, we can translate constraints in the relative variance into constraints in the stability function. Since we are interested in constructing explicit methods we can ask how we can make *ψ*(*z*) close to 1 in an explicit framework for which we already know the stability function is a polynomial of at most degree s (equation (6))

$$R(z)=1+\sum_{j=1}^{s}r_{j}z^{j}.$$

Thus, similar to the case *s *= 2 in which we had one free parameter, *γ*, to optimise, if we assume *r*_1 _= **b**^T^**e **= 1 then we have *s *- 1 parameters, *r*_2_,...,*r*_*s*_, we can optimise. In this case, though, the search of the optimal set of parameters has to be performed with numerical optimisation methods rather than analytically. The problem of finding optimal sets of parameters can be stated as a nonlinear program, NLP, and thus its solution approximated numerically (see details in Additional file 1).

**Figure 1:**
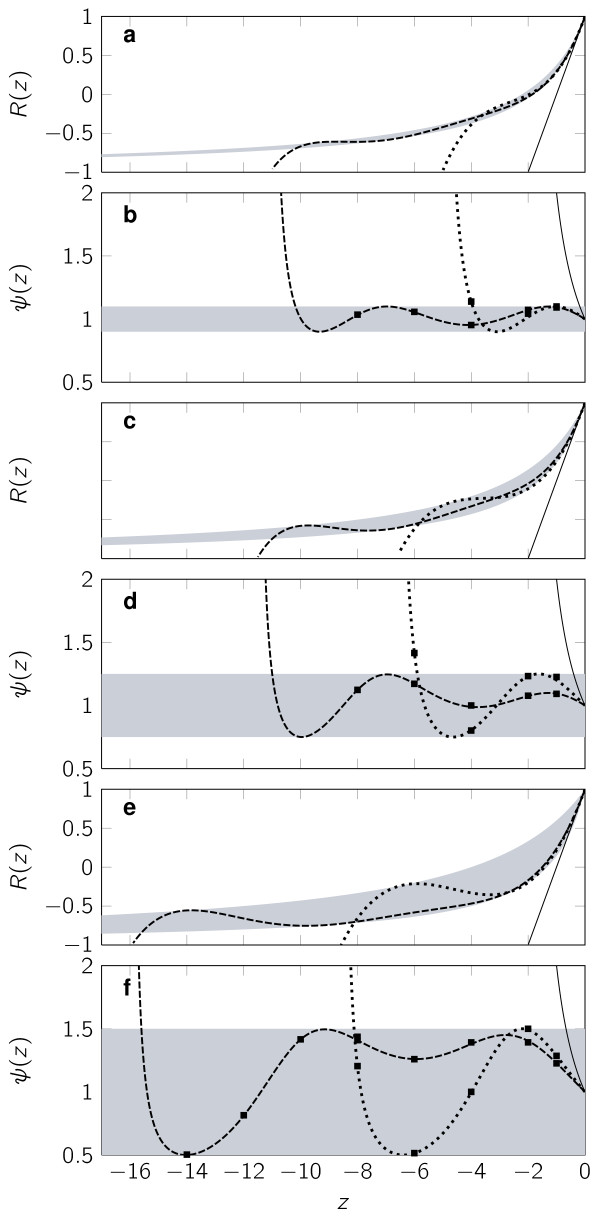
**Stability and relative variance for the different methods**. Stability and relative variance functions for the Poisson *τ*-leap method (solid line) and RK *τ*-leap methods with optimal stability regions and bounded relative variance (*ψ*) with 3 stages (dotted line) and 5 stages (dashed line). Regions fulfilling the bounds on *ψ *are shown in grey. Square dots correspond to relative variances computed from 10^6 ^simulations each. **(a)**, **(b)**: Relative variance bounded by 0.1. **(c)**, **(d)**: Relative variance bounded by 0.25. **(e)**, **(f)**: Relative variance bounded by 0.5.

Figure 1 shows the stability function and relative variance function for the Poisson *τ*-leap, and optimal methods for *s *= 3 and *s *= 5 under the constraints |*ψ*(*z*)-1| < 0.1, 0.25 and 0.5 and Table 1 summarises the numerical values for these conditions.

**Table 1:** Stability regions for methods with bounded relative variance and optimal stability

Bound (ϵ)	Stages (*s*)	**Stability (*l***_***s*, ϵ**_**)**	Factor vs. *τ*-leap	Norm. factor *τ*-leap
0.10	3	3.94566	19.73	6.58
	5	10.1813	50.9	10.18

0.25	3	5.89563	14.74	4.91
	5	11.0001	27.5	5.5

0.50	3	8.12004	12.18	4.06
	5	15.5997	23.4	4.68

Factor vs. *τ*-leap corresponds to the relative increase in the step size with respect to the Poisson *τ*-leap with the same bound in the variance (*l*_*s*, ϵ_/*l*_*τ*-leap_). Normalised factor vs. *τ*-leap takes into account the multiple stages of the method (*l*_*s*, ϵ_/(*s*·*l*_*τ*-leap_)).

#### Efficient methods with bounded variance and extended stability

Runge-Kutta methods with a given stability polynomial *R*(*z*) are not unique. This is because the stability polynomial only reflects the application of a Runge-Kutta method to a linear problem. Nonlinear problems require many additional order conditions to be satisfied in order for a method to have a certain order of accuracy. Thus many different methods can have the same stability polynomial. Furthermore, we have already seen that the relative variance *ψ *does not directly depend on **A **but on *R*(*z*) thus making all methods with the same stability function behave identically in terms of stationary variance for linear problems. In order to distinguish between methods with the same stability function we would have to consider more complicated nonlinear chemistry and this is beyond the scope of this work. However, we have an explicit way of constructing an efficient method that has a given stability polynomial (i.e. to find values for **b **and **A **of the Butcher tableau, see details in Additional file 1). Furthermore, the tableaus build in this way are such that *β*_*s *_= 1, *β*_j _= 0, *j *= 0,...,*s *- 1 and **A **has all its elements set to zero except those on the first subdiagonal. These Runge-Kutta schemes obtained in this way are very natural, can be regarded as fixed point iterations and allow the following efficient reformulation of (17)

(29) $$\begin{array}{c} Y_{1}=y_{n}\\ Y_{i}=y_{n}+\alpha_{i,i-1}(\tau f(Y_{i-1})+d_{n}),\text{for }i=2,\ldots ,s\\ y_{n+1}=y_{n}+\tau f(Y_{s})+d_{n}. \end{array}$$

It is thus clear that these methods are computationally more efficient than the general case as they only require *s*-1 evaluations of the expected step-change **f**(·) instead of the *s*(*s *- 1)/2 required in the general framework (17). A collection of methods have been implemented in a branch of the ByoDyn package, v.5.0 [39].

### Numerical results

#### Reversible isomerisation

**Figure 2:**
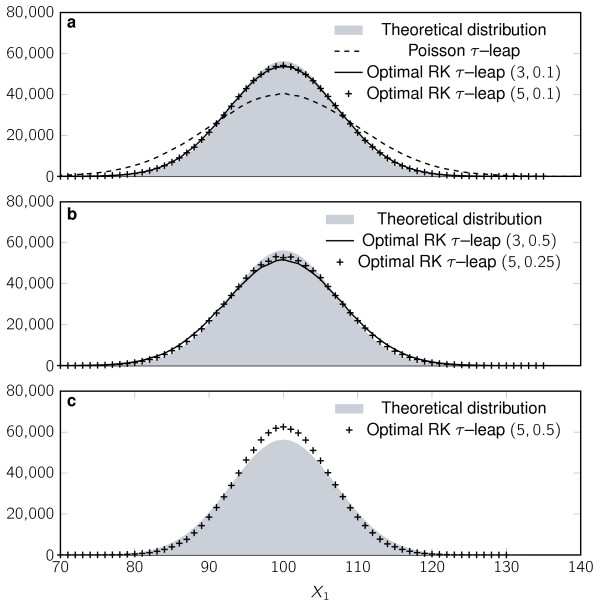
**Histogram of *X*_1 _in the Reversible isomerisation reaction**. Histogram of *X*_1 _in the Reversible isomerisation reaction (10^6 ^samples used) solved by the SSA (grey background), Poisson *τ*-leap (dashed line), and optimal RK *τ*-leap methods with bounded relative variance. **(a) ***τ *= 0.05 (*z *= -1), Optimal RK *τ*-leap *s *= 3, ϵ = 0.1 (solid line) and *s *= 5, ϵ = 0.1 ("+" marks), **(b) ***τ *= 0.4 (z = -8), Optimal RK *τ*-leap *s *= 3, ϵ = 0.5 (solid line) and *s *= 5, ϵ = 0.1 ("+" marks), Poisson *τ*-leap is unstable for this time step. **(c) ***τ *= 0.6 (*z *= -12), Optimal RK *τ*-leap *s *= 5, ϵ = 0.5 ("+" marks), Poisson *τ*-leap and Optimal RK *τ*-leap *s *= 3 are unstable for this time step.

We compare the new Runge-Kutta framework to the Poisson *τ*-leap to solve three systems of chemical reactions. The first is the reversible isomerisation test problem in (21) for which we have already developed theoretical results. Numerical simulation of the number of molecules for each of the two components in the system was carried out using the different methods discussed in the previous section with *k*_1 _= *k*_2 _= 10 (z = -20*τ*) and **X**(0) = (100, 100)^T^. We sampled 10^6 ^trajectories for each of the methods and for different fixed *τ *values. Figure 2 shows a comparison between the true probability density function (PDF) and the histograms of *X*_1 _obtained from the different methods and some of the values of *τ*. Note that the Poisson *τ*-leap method becomes unstable for *τ *> 0.1 and so does RK *τ*-leap with three stages for *τ *> 0.4. Figure 1 shows that the stationary variances obtained by the simulations are in exact accordance with the theoretical values derived in the previous section.

#### Schlögl reaction

**Figure 3:**
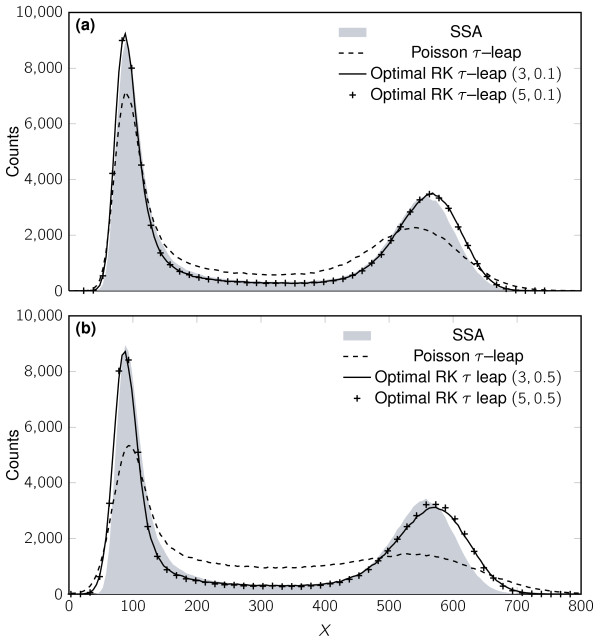
**Histogram of *X *in the Schlögl reaction**. Histogram of *X *in the Schlögl reaction (10^6 ^samples used) solved by the SSA (grey background), Poisson *τ*-leap (dashed line), and Optimal RK *τ*-leap methods. **(a) ***τ *= 0.4, Optimal RK *τ*-leap *s *= 3, ϵ = 0.1 (solid line) and *s *= 5, ϵ = 0.1 ("+" marks), **(b) ***τ *= 0.8, Optimal RK *τ*-leap *s *= 3, ϵ = 0.5 (solid line) and *s *= 5, ϵ = 0.5 ("+" marks).

**Figure 4:**
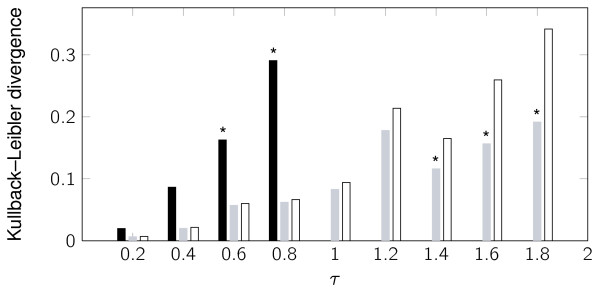
**Kullback-Leibler divergence for the Schlögl reaction**. Kullback-Leibler divergence between the exact stationary distribution of *X *in the Schlögl reaction (estimated by 10^6 ^samples solved by SSA) and the approximate stationary distributions obtained with the Poisson *τ*-leap (black), Optimal RK *s *= 3; ϵ = 0.5 (grey lines) and Optimal RK *s *= 5, ϵ = 0.5 (white). Bars are shown only for the stable method and *τ *settings. Asterisks denote methods that have a rate of failure above 10^-3^.

We also consider Schlögl's autocatalytic reaction system [34,40] to illustrate the accuracy of the presented framework, developed for the linear case, for nonlinear systems. We use here the same set of parameters as Cao et al. [38] for which this system presents a bimodal PDF for the species *X *in the stationary state. We have also considered that the non-autocatalytic species are buffered (assuming they are constant) hence reducing the system to a scalar problem (see Table 2). We have again performed 10^6 ^simulations for each method and *τ *value. Figure 3 shows histograms computed by the SSA, Poisson *τ*-leap and the methods with *s *= 3, 5. Visual inspection of the plots shows a consistent improvement over the original *τ*-leap method by means of the multistage RK methods developed here. **A **more precise comparison of the plots is given in Figure 4, which shows the estimated Kullback-Leibler divergences between the exact PDF (*P*_E_) and the PDFs of each of these methods (*P*_M_), given by:

**Table 2:** Details of the Schlögl reaction system

Reactions	Parameters	Propensities
$A+2X\overset{k_{1}}{\to }3X$	*k*_1 _= 3·10^-7^	*k*_1_*x*(*x *- 1)*A*/2
$3X\overset{k_{2}}{\to }A+2X$	*k*_2 _= 10^-4^	*k*_2_*x*(*x *- 1)(*x *- 2)/6
$X\overset{k_{3}}{\to }B$	*k*_3 _= 3.5	*k*_3_*x*
$B\overset{k_{4}}{\to }X$	*k*_4 _= 10^-3^	*k*_4_*B*

(30) $$D(P_{\text{E}},P_{\text{M}})=\sum_{x}P_{\text{E}}(x)\log_{2}\left(\frac{P_{\text{E}}(x)}{P_{\text{M}}(x)}\right).$$

#### The MAPK cascade

Finally, we have tested the performance of our methods on a larger system of chemical reactions with stiffness due to different reaction time scales and species amounts ranging over several orders of magnitude. For this purpose we considered the Huang and Ferrell model for the mitogen-activated protein kinase (MAPK) cascade [41]. This model is available from the BioModels database [42] and consists of 22 species interacting through 30 reaction channels. The set of parameters used here (see Additional file 1 for details) renders the model stiff and with species amounts ranging from none up to 3·10^5 ^molecules. With the chosen initial conditions the system undergoes a transient change and finally settles down into a stationary state at around *t *= 150 minutes. We have simulated the model using SSA (Gillespie's Direct Method), the Poisson *τ*-leap and the RK methods presented here. To produce fair comparisons, all methods have been rewritten in ANSI C using the Mersenne twister [43] pseudorandom number generator from the GNU Scientific Library. The GNU C Compiler was used to compile the sources with the -O2 optimisation flag. The algorithms were run on an Intel(R) Core(TM)2 Duo Processor E8500 at 3.16 GHz and 6 MB cache. We have run the system to a final time T = 200. Simulations run with SSA took 61, 841 ± 74 seconds.

We have compared the methods in two distinct situations. First we have run them with the same time step *τ *= 5·10^-5^. In this case, the Poisson *τ*-leap method took 51.7 ± 0.4 seconds while Optimal RK *τ*-leap methods with *s *= 3 and *s *= 5 took 86.1 ± 0.4 seconds and 113.9 ± 0.3 seconds respectively. Hence, at the same time step the RK methods are approximately 66% and 120% slower than the Poisson *τ*-leap due to the multiple evaluations of the propensity functions per step. However, there is an important difference in the results. The relative variance at the steady state is 1.3 (see Additional file 1) for the Poisson *τ*-leap while for both RK *τ*-leap methods with *s *= 3 and *s *= 5 (ϵ = 0.1) it is less than 1.04.

Then we have compared these methods when run at their respective maximum time steps such that the relative variance at the stationary state is bounded to 1.1 (estimated from the simulations). The maximum time steps allowed with this constraint were: *τ *= 2·10^-5 ^for the Poisson *τ*-leap, *τ *= 3.5·10^-4 ^for the RK *τ*-leap (3, 0.1) and *τ *= 9.5·10^-4 ^for the Optimal RK *τ*-leap (5, 0.1). With this setting, the runtimes obtained were: 111.9 ± 0.7 seconds for the Poisson *τ*-leap, 15.7 ± 0.06 seconds for the RK *τ*-leap (3, 0.1) and 7.8 ± 0.02 seconds for the Optimal RK *τ*-leap (5, 0.1). Thus, in this case the Poisson *τ*-leap approximately 7.1 and 14.3 times slower than the RK methods, respectively.

## Discussion

Biochemical kinetics typically deals with multiscale problems, in which several scales of time, space and concentrations, simultaneously affect the dynamical behaviour of the system. Thus, the systems biology community is deeply interested in the development of methods that lead to a multiscale view of biochemical systems. As a first step in this workflow, we have presented here a new set of methods that considerably expands the classical *τ*-leap implementation, from a stability perspective. The importance of the results shown here embraces not only the increase in computational speed for stochastic simulations, a key element for the understanding of the intrinsically noisy biological systems, but more importantly, a way to deal with fast reactions in multiscale settings. The methods developed here have been demonstrated for a first example of stiff system, the classical Schlögl autocatalytic reaction, and can be straightforwardly incorporated into hybrid SSASDE-ODE frameworks.

We see from Table 1 that if we require a bound on the equilibrium variance of 0.1 then the Poisson *τ*-leap method must take $\left|\underset{\cdot }{z}\right|\leq \frac{2}{11}$ while for the RK methods the bounds on |*z*| are approximately 4 and 10, respectively with *s *= 3, 5. This is a very considerable improvement and all the more striking given that the same number of Poisson random variables are simulated per step in all cases.

Initially we had hoped that an approach via Chebyshev methods using ideas from ODEs and SDEs applied to the discrete cases would have been fruitful. It turns out that while such methods have good mean behaviour, the variance behaviour is poor. This is because the variance growth function satisfies (24) and an s-stage Chebyshev method would have *s *- 1 poles and zeros due to the oscillations in the stability function. Similar issues arise even in the damped forms of the Chebyshev formulation. This means that our optimisation approach is the only way of getting good bounds on *ψ*(*z*).

Our results on the nonlinear bimodal Schlögl problem show that the RK methods still behave appropriately even on nonlinear problems. For example, from Figure 3 we see that the Poisson *τ*-leap method is not very accurate with *τ *= 0.4 and quite poor in picking up the second peak with *τ *= 0.8. On the other hand the RK methods match the peak quite well, albeit with a slight shift in that peak. Furthermore, numerical results from the MAPK cascade simulations show that our methods can run an order of magnitude faster than the Poisson *τ*-leap and still give the same accuracy in the results.

Finally, we note that we could extend our RK methods to allow more than one set of Poisson random variables to be simulated per step. We imagine that this would allow even bigger stepsizes but at the cost of taking more simulation time in that the additional Poisson sampling is expensive. We emphasise that although our analysis of these new methods has been given for unimolecular reactions, the simulations of the nonlinear Schlögl reaction and the MAPK cascade indicate that these methods have a more general applicability and we will consider nonlinear analysis via Taylor series expansions in future work.

## Authors' contributions

KB, PR and JVF designed the research. KB and PR developed the algorithms and PR implemented them and performed and analysed the simulations. KB, PR and JVF wrote the manuscript. All authors have read and approved the final version of the manuscript.

## Supplementary Material

Additional file 1 **Supplementary material for "Simulation methods with extended stability for stiff biochemical kinetics"**. Technical results, coefficients for the optimal stability polynomials and notes on the mitogen-activated protein kinase (MAPK) cascade simulation results. Click here for file
