# Supplementary material for: Simulation methods with extended stability for stiff biochemical Kinetics
Source: BMC Syst Biol. 2010 Aug 11;4:110. doi: 10.1186/1752-0509-4-110 (PMC3225827; doi:10.1186/1752-0509-4-110)
Supplement: Additional file 1 — Supplementary material for "Simulation methods with extended stability for stiff biochemical kinetics". Technical results, coefficients for the optimal stability polynomials and notes on the mitogen-activated protein kinase (MAPK) cascade simulation results. [file 1752-0509-4-110-S1.PDF]

Supplementary material for "Simulation methods with extended stability for stiff biochemical kinetics"

Pau Rué[1,2] , Jordi Villà–Freixa*[1] , Kevin Burrage*[3,4]

[1]Computational Biochemistry and Biophysics Group, Research Unit on Biomedical Informatics, IMIM / Universitat Pompeu Fabra, c/ Dr. Aiguader 88, 08003, Barcelona, Catalonia, Spain
[2]Current address: Departament de Física i Enginyeria Nuclear, Universitat Politècnica de Catalunya, Edifici GAIA, Rambla de Sant Nebridi s/n 08222, Terrassa, Barcelona, Spain
[3]Institute for Molecular Bioscience, The University of Queensland, Brisbane, QLD, 4072, Australia
[4]COMLAB and OCISB, University of Oxford, Oxford OX1 3QD, United Kingdom

Email: Pau Rué - pau.rue@upc.edu; Jordi Villà–Freixa*- jordi.villa@upf.edu; Kevin Burrage*- kevin.burrage@comlab.ox.ac.uk;

*Corresponding author

Technical results

Mean and variance behaviour of the framework

In order to analyse the mean and variance behaviour of

$$\mathbf{d}_n = \sum_{j=1}^{m} \nu_j L\left(\tau, a_j(\mathbf{X}_n)\right) \tag{1}$$

$$\mathbf{Y}_i = \mathbf{X}_n + \tau \sum_{j=1}^{i-1} \alpha_{ij} \mathbf{f}(\mathbf{Y}_j) + \omega_i \mathbf{d}_n, \quad i = 1, \dots, s$$

$$\mathbf{X}_{n+1} = \mathbf{X}_n + \tau \sum_{j=1}^{s} \beta_j \mathbf{f}(\mathbf{Y}_j) + \mathbf{d}_n$$

when applied to a general set of m unimolecular reactions can be described by m propensity functions given by the following linear functions

$$a_j(\mathbf{x}) = \sum_{i=1}^{N} c_{ij} x_i = \mathbf{c}_j{}^{\mathsf{T}} \mathbf{x}, \quad j = 1, \dots, m, \tag{2}$$

the following Lemma on conditional random variables will prove vital

Lemma 1. *If X and Y are random variables then*

$$\mathbb{E}\left[Y\right] = \mathbb{E}\left[\mathbb{E}\left[Y|X\right]\right]$$

$$Var\left[Y\right] = \mathbb{E}\left[Var\left[Y|X\right]\right] + Var\left[\mathbb{E}\left[Y|X\right]\right].$$

Lemma 2.

$$\mathbb{E}\left[\mathcal{P}\left(\tau\lambda X\right)\right] = \tau\lambda X, \quad \mathit{Var}\left[\mathcal{P}\left(\tau\lambda X\right)\right] = \tau\lambda X.$$

Using these two Lemmas we now prove the following theorems.

Theorem 3. *Let the Runge-Kutta method underlying a Runge-Kutta τ-leap method have stability function $R(z) = 1 + \mathbf{b}^{\mathsf{T}} z(\mathbf{I} - \mathbf{A}z)^{-1}\mathbf{e}$, then when* (1) *is applied to* (2)

$$\mathbb{E}\left[\mathbf{X}_{n+1}\right] = R(\tau\mathbf{W})\mathbb{E}\left[\mathbf{X}_n\right], \tag{3}$$

where $\mathbf{e}$ *is the unit vector and*

$$R(\tau\mathbf{W}) = \mathbf{I}_N + \tau\mathbf{b}^{\mathsf{T}} \otimes \mathbf{W}\left(\mathbf{I}_s \otimes \mathbf{I}_N - \tau\mathbf{A} \otimes \mathbf{W}\right)^{-1}\left(\mathbf{e} \otimes \mathbf{I}_N\right).$$

Proof. Let $\mathbf{Y} = (\mathbf{Y_1}, \ldots, \mathbf{Y_n})^{\mathsf{T}}$, applying (1) to (2), noticing that $\mathbf{f}(\mathbf{x}) = \mathbf{W}\mathbf{x}$ and using the first equations in Lemma 1 and 2:

$$\begin{aligned}
\mathbb{E}\left[\mathbf{Y}\right] &= \mathbf{e}_s \otimes \mathbb{E}\left[\mathbf{X}_n\right] + \tau\mathbf{W} \otimes \mathbf{A}\mathbb{E}\left[\mathbf{Y}\right], \\
\mathbb{E}\left[\mathbf{Y}\right] &= (\mathbf{I}_s \otimes \mathbf{I}_N - \tau\mathbf{W} \otimes \mathbf{A})^{-1}\mathbf{e}_s \otimes \mathbb{E}\left[\mathbf{X}_n\right], \\
\mathbb{E}\left[\mathbf{X}_{n+1}\right] &= \mathbb{E}\left[\mathbf{X}_n\right] + \tau\mathbf{b}^{\mathsf{T}} \otimes \mathbf{W}\mathbb{E}\left[\mathbf{Y}\right],
\end{aligned}$$

the result follows immediately. □

Corollary 4. *If* (1) *is applied to the reversible isomerisation reaction with fixed total number of molecules*

$$S_1 \underset{k_2}{\overset{k_1}{\rightleftarrows}} S_2, \tag{4}$$

defined by

$$\mathbf{W} = \begin{pmatrix} -k_1 & k_2 \\ k_1 & -k_2 \end{pmatrix}. \tag{5}$$

with constant τ such that $|R(z)| < 1$, $z = -\tau(k_1 + k_2)$, then in the limit as $n \to \infty$ the mean vector converges to the theoretical mean, that is

$$\lim_{n\to\infty} \mathbb{E}\left[\mathbf{X}_n\right] = \mathbb{E}\left[\mathbf{X}^*\right] = \frac{T}{k_1 + k_2}(k_2, k_1)^{\mathsf{T}}. \tag{6}$$

Proof. We can write

$$\mathbf{W} = \mathbf{V}\mathrm{Diag}\left(0, -(k_1 + k_2)\right)\mathbf{V}^{-1}$$

with

$$\mathbf{V} = \begin{pmatrix} k_2 & 1 \\ k_1 & -1 \end{pmatrix}.$$

Hence

$$R(\tau\mathbf{W}) = \mathbf{V}\text{Diag}\left(R(0), R(-\tau(k_1+k_2))\right)\mathbf{V}^{-1}$$
$$= \mathbf{V}\text{Diag}\left(1, R(-\tau(k_1+k_2))\right)\mathbf{V}^{-1}.$$

Thus

$$R(\tau\mathbf{W})^n = \mathbf{V}\text{Diag}\left(1, R^n(-\tau(k_1+k_2))\right)\mathbf{V}^{-1}$$

and with τ such that $|R(-\tau(k_1+k_2))| < 1$, then

$$\lim_{n\to\infty} R^n(-\tau(k_1+k_2)) = 0$$

so that

$$\begin{aligned}\lim_{n\to\infty} \mathbb{E}\left[\mathbf{X}_n\right] =& \mathbf{V}\text{diag}\left(1,0\right)\mathbf{V}^{-1}\mathbb{E}\left[\mathbf{X}_0\right] \\ =& \frac{1}{k_1+k_2}\begin{pmatrix} k_2 & k_2 \\ k_1 & k_1 \end{pmatrix}\mathbb{E}\left[\mathbf{X}_0\right] \\ =& \frac{T}{k_1+k_2}\begin{pmatrix} k_2 \\ k_1 \end{pmatrix},\end{aligned}$$

and the result holds from the properties of the binomial distribution with $\mathbf{e} = (1,1)^{\mathsf{T}}$

$$\begin{aligned}\mathbb{E}\left[\mathbf{X}^*\right] &= \frac{T}{k_1+k_2}(k_2,k_1)^{\mathsf{T}} \\ \text{Var}\left[\mathbf{X}^*\right] &= \frac{T}{(k_1+k_2)^2}k_1k_2\mathbf{e}.\end{aligned} \tag{7}$$

□

Theorem 5. *If the coefficients in the underlying Runge-Kutta method in* (1) *are non–negative, and if* (1) *is applied to* (5) *with constant* τ *then in the limit as* $n \to \infty$*, writing* $Var\left[\mathbf{X}_\infty\right] = \lim_{n\to\infty} Var\left[\mathbf{X}_n\right]$ *then*

$$Var\left[\mathbf{X}_\infty\right] = \frac{2}{z}\left(\frac{R(z)-1}{R(z)+1}\right) Var\left[\mathbf{X}^*\right],$$

where $Var\left[\mathbf{X}^*\right]$ *is given by* (7) *and*

$$R^2(z) \neq 1, \quad z = -\tau(k_1+k_2).$$

Proof. If we apply (1) to (5) and let y_n and x_n be the update numbers of S_1 and S_2 and Y_{in} and X_{in} $(i = 1,\ldots,s)$ be the intermediate numbers of S_1 and S_2, respectively, then it is clear that

$$y_n + x_n = Y_{in} + X_{in} = T, \quad \forall n.$$

Thus we can reduce the problem to a one dimensional problem and also note that $\mathrm{Var}\left[y_n\right] = \mathrm{Var}\left[x_n\right], \forall n$. Thus, in this case, the method becomes

$$Y_{in} = y_n + \tau \sum_{j=1}^{m} \alpha_{ij}(S - KY_{jn}) + \omega_i d_n$$
$$y_{n+1} = y_n + \tau \sum_{j=1}^{m} \beta_j (S - KY_{jn}) + d_n$$

with

$$\nu_1 = -1 \qquad a_1(x) = k_1 x$$
$$\nu_2 = +1 \qquad a_2(x) = k_2(T - x)$$
$$z = -\tau K \qquad d_n = -L\left(\tau, k_1 y_n\right) + L\left(\tau, k_2(T - y_n)\right).$$

Using vector notation, $\mathbf{Y}_n = (Y_{1n}, \ldots, Y_{sn})^{\mathsf{T}}, \quad \mathbf{e} = (1, \ldots, 1)^{\mathsf{T}}$, and assuming $\mathbf{w} = \mathbf{A}\mathbf{e}$

$$\mathbf{Y}_n = \mathbf{e}y_n + \mathbf{A}\mathbf{e}(d_n + \tau S) + z\mathbf{A}\mathbf{Y}$$
$$\mathbf{Y}_n = (\mathbf{I} - z\mathbf{A})^{-1}(\mathbf{e}y_n + \mathbf{A}\mathbf{e}\tau S + \mathbf{A}\mathbf{e}d_n) \tag{8}$$
$$y_{n+1} = y_n + \tau(\mathbf{b}^{\mathsf{T}}\mathbf{e}S - K\mathbf{b}^{\mathsf{T}}\mathbf{Y}) + d_n \tag{9}$$
$$= y_n(1 + z\mathbf{b}^{\mathsf{T}}(\mathbf{I} - z\mathbf{A})^{-1}\mathbf{e}) + (\tau S + d_n)(1 + z\mathbf{b}^{\mathsf{T}}(\mathbf{I} - z\mathbf{A})^{-1}\mathbf{A}\mathbf{e}).$$

But $\tau S + d_n = zy_n + \mathcal{P}\left(\tau k_2(T - y_n)\right) - \mathcal{P}\left(\tau k_1 y_n\right)$

$$y_{n+1} = y_n\left(1 + z\mathbf{b}^{\mathsf{T}}(\mathbf{I} - z\mathbf{A})^{-1}\mathbf{e} + z\left(1 + z\mathbf{b}^{\mathsf{T}}(\mathbf{I} - z\mathbf{A})^{-1}\mathbf{A}\mathbf{e}\right)\right)$$
$$+ \left(\mathcal{P}\left(\tau k_2(T - y_n)\right) - \mathcal{P}\left(\tau k_1 y_n\right)\right)\left(1 + z\mathbf{b}^{\mathsf{T}}(\mathbf{I} - z\mathbf{A})^{-1}\mathbf{A}\mathbf{e}\right)$$
$$= y_n\left(1 + z - z\mathbf{b}^{\mathsf{T}}(\mathbf{I} + z\mathbf{A})^{-1}(\mathbf{e} + z\mathbf{A}\mathbf{e})\right)$$
$$+ \left(\mathcal{P}\left(\tau k_2(T - y_n)\right) - \mathcal{P}\left(\tau k_1 y_n\right)\right)\left(1 + z\mathbf{b}^{\mathsf{T}}(\mathbf{I} - z\mathbf{A})^{-1}\mathbf{A}\mathbf{e}\right)$$
$$y_{n+1} = y_n + \frac{R(z) - 1}{z}\left(\mathcal{P}\left(\tau k_2(T - y_n)\right) - \mathcal{P}\left(\tau k_1 y_n\right)\right), \tag{10}$$

with $(\mathbf{I} - z\mathbf{A})^{-1}\mathbf{A} = z^{-1}((\mathbf{I} - z\mathbf{A})^{-1} - \mathbf{I})$. Applying Lemma 1 we get

$$\mathrm{Var}\left[y_{n+1}\right] = \mathrm{Var}\left[\mathbb{E}\left[y_{n+1}|y_n\right]\right] + \mathbb{E}\left[\mathrm{Var}\left[y_{n+1}|y_n\right]\right]$$
$$= \mathrm{Var}\left[R(z)y_n\right] + \left(\frac{R(z) - 1}{z}\right)^2 \mathbb{E}\left[k_2\tau(T - y_n) + k_1\tau y_n\right]$$
$$\mathrm{Var}\left[y_{n+1}\right] = R(z)^2\mathrm{Var}\left[y_n\right] + \left(\frac{R(z) - 1}{z}\right)^2 (k_2\tau T + \tau(k_1 - k_2)\mathbb{E}\left[y_n\right]). \tag{11}$$

Let $n \to \infty$ and use (6) then

$$k_2 \tau T + \tau(k_1 - k_2)\frac{k_2 T}{k_1 + k_2} = \frac{k_2 \tau T}{k_1 + k_2}(2k_1) = \frac{2k_1 k_2 \tau T}{k_1 + k_2} = -2z\mathrm{Var}\left[y^*\right].$$

So with $|R(z)| < 1$

$$\begin{aligned}\mathrm{Var}\left[y_\infty\right] &= \frac{2z}{z^2}\frac{(R(z)-1)^2}{R(z)^2-1}\mathrm{Var}\left[y^*\right] = \frac{2}{z}\frac{R(z)-1}{R(z)+1}\mathrm{Var}\left[y^*\right] \\ &= \psi(z)\mathrm{Var}\left[y^*\right].\end{aligned} \tag{12}$$

□

Remark 6. The factor by which the true variance of (5) at the stationary state is modified when of a Runge-Kutta method with stability function $R(z)$ (1) is applied to (5) is given by

$$\psi(z) = \frac{2}{z}\frac{R(z)-1}{R(z)+1}, \tag{13}$$

where $|R(z)| < 1$. In deriving this result we have assumed

$$\mathbf{b}^{\mathsf{T}}\mathbf{e} = 1, \quad \mathbf{A}\mathbf{e} = \mathbf{w}$$

and all the coefficients in (1) are non–negative.

Optimisation of the coefficients of the methods

Once we have defined the framework and computed the stability function and the relative variance function we want to design particular explicit methods with bounded variance and extended stability domain. As we are considering explicit methods for which the stability function becomes a polynomial of degree at most s, this is equivalent to finding the optimal values for the parameters r_j, $j = 2, \dots, s$ $R(z)$ in

$$R(z) = 1 + \sum_{j=1}^{s} r_j z^j. \tag{14}$$

Thus, by substituting (14) in (13)

$$\begin{aligned}\psi(z) &= \frac{1 + \sum_{j=1}^{s-1} r_{j+1} z^j}{1 + \frac{z}{2} + \sum_{j=2}^{s} \frac{r_j}{2} z^j} \\ &= 1 + \frac{(r_2 - \frac{1}{2})z + \sum_{j=2}^{s-1}(r_{j+1} - \frac{r_j}{2})z^j - \frac{r_s}{2}z^s}{1 + \frac{z}{2} + \sum_{j=2}^{s} \frac{r_j}{2} z^j} \\ &= 1 + \frac{N(z)}{D(z)}.\end{aligned} \tag{15}$$

Now then consider the $s-1$–dimensional vector $\mathbf{r} = (r_s, \dots, r_2)^\mathsf{T}$ of coefficients defining the polynomial $R(z) = R(z, \mathbf{r})$ and the rational function $\psi(z) = \psi(z, \mathbf{r}) = 1 + N(z, \mathbf{r})/D(z, \mathbf{r})$. Note that the notation $R(z, \mathbf{r})$ just represents the stability function with free parameters r_j, $j = 2, \dots, s$.

The problem of finding stability functions for methods with maximum stability region and bounded variance can be stated as a Nonlinear Program (NLP). Let us consider, if it exists, the real negative pole closest to zero, $\pi(\mathbf{r})$, of $\psi(z, \mathbf{r})$, otherwise, we set $\pi(\mathbf{r}) = -\infty$. In the region $(\pi(\mathbf{r}), 0)$ ψ is a continuous rational function and thus the value of l_s can be derived analytically taking into account its local extrema in this region.

Let $\rho(\mathbf{r})$ be the set of all real roots of the polynomial $\frac{\mathrm{d}}{\mathrm{d}z} N(z, \mathbf{r}) D(z, \mathbf{r}) - N(z, \mathbf{r}) \frac{\mathrm{d}}{\mathrm{d}z} D(z, \mathbf{r})$ in $(\pi(\mathbf{r}), 0)$ and let $\kappa(\mathbf{r})$ be the set of all real roots of either $N(z, \mathbf{r}) - \epsilon D(z, \mathbf{r})$ or $N(z, \mathbf{r}) + \epsilon D(z, \mathbf{r})$ which lie in the region $(\pi(\mathbf{r}), 0)$.

We can define the boundary functions B_U and B_L as

$$B_U(\mathbf{r}) = \max_{s \in \rho(\mathbf{r})} \{\psi(s, \mathbf{r})\}, \quad B_L(\mathbf{r}) = \min_{s \in \rho(\mathbf{r})} \{\psi(s, \mathbf{r})\} \tag{16}$$

and the objective function $l_s(\mathbf{r})$ as the left most element of $\kappa(\mathbf{r})$.

$$\max\ l_s(\mathbf{r}) : \qquad \mathbf{r} \in [0,1]^{s-1} \quad B_U(\mathbf{r}) - 1 - \epsilon < 0 \quad B_L(\mathbf{r}) - 1 + \epsilon > 0. \tag{17}$$

Efficient methods

Once we have determined the optimal stability polynomial of order s for a certain given ϵ bound, $R(z)$, we can ask how to construct a method (i.e., how to find $\mathbf{b}$ and $\mathbf{A}$) with such polynomial as stability function. We describe now how to construct and implement the simplest and fastest explicit method of a given stability polynomial. This can be accomplished by having all the elements of $\mathbf{A}$ zero except for nonzeros on the first subdiagonal, as the following theorem shows.

Theorem 7. *Given the coefficients, $r_2, \dots, r_s > 0$ there always exist at least one fixed–point iteration method with Butcher tableau $(\mathbf{A}, \mathbf{b})$ such that $r_j = \mathbf{b}^\mathsf{T} \mathbf{A}^{j-1} \mathbf{e}$. In particular,*

$$\alpha_{ij} = \begin{cases} r_2, & \textit{if } i = s, j = s-1, \\ r_{j+2}/r_{j+1}, & \textit{if } j = 1, \dots, s-2,\ i = s-j, \\ 0, & \textit{otherwise} \end{cases}$$

and $\mathbf{b} = (0, \dots, 0, 1)^\mathsf{T}$ fulfils this condition.

Proof. Let $\mathbf{A} = (\alpha_{ij})$ be a strictly lower triangular matrix with all coefficients but $\alpha_{i+1,i}$ set to zero. Denote by $\alpha_{ij}^{(k)}$ the coefficients of the matrix $\mathbf{A}^k$, $k = 2, \dots, s-1$. Obviously $\mathbf{A}^k = 0$ for $k \geq s$. The coefficients $\alpha_{ij}^{(k)}$ are all zero but for $\alpha_{i+k,i}^{(k)}$, $i = 1, \dots s-k$, which take the values $\alpha_{i+k,i}^{(k)} = \prod_{j=1}^{k} \alpha_{i+j,i+j-1}$. On the other hand, let $\mathbf{b} = (0, \dots, 0, 1)^\top$, then, for any $s \times s$ matrix $\mathbf{M} = (m_{ij})$, $\mathbf{b}^\top \mathbf{M} \mathbf{e} = \sum_{j=1}^{s} m_{sj}$. In particular, for $\mathbf{M} = \mathbf{A}^{(k)}$,

$$\mathbf{b}^\top \mathbf{A}^k \mathbf{e} = \alpha_{s,s-k}^{(k)} = \prod_{j=1}^{k} \alpha_{s-k+j,s-k+j-1} = \alpha_{s-k+1,s-k} \cdots \alpha_{s,s-1}.$$

Thus, we can obtain $\mathbf{A}$ by solving $r_j = \alpha_{s-j+2,s-j+1} \cdots \alpha_{s,s-1}, j = 2, \dots, s$ and hence

$$\alpha_{s,s-1} = r_2$$

$$\alpha_{s-j,s-j-1} = r_{j+2}/r_{j+1}, \quad j = 1, \dots, s-2.$$

□

Note that this construction is not unique and that there are other formulations which would lead to methods with the same stability function.

Coefficients for the optimal stability polynomials

Table shows the values of the coefficients of the optimal stability polynomials $R(z)$ with bounded stationary variance and the coefficients of the corresponding fixed-point iteration Butcher tableaus $(\mathbf{A}, \mathbf{b})$.

**Stages** s	**Bound** ϵ	**Stability** l_s	$R(z)$ **Coefficients**	**Butcher tableau coefficients coefficients**
3	0.10	3.94566	r_3 = 4.240285321762531e-02	a_{21} = 1.274094895306793e-01
			r_2 = 3.328076532903382e-01	a_{32} = 3.328076532903382e-01
	0.25	5.89563	r_3 = 2.561709328231343e-02	a_{21} = 9.302165557301426e-02
			r_2 = 2.753884901801833e-01	a_{32} = 2.753884901801833e-01
	0.50	8.12004	r_3 = 1.692030482039519e-02	a_{21} = 7.215758807926195e-02
			r_2 = 2.344909977008790e-01	a_{32} = 2.344909977008790e-01
5	0.10	10.1813	r_5 = 2.259761095046997e-04	a_{21} = 3.512099547699939e-02
			r_4 = 6.434217095375061e-03	a_{32} = 9.333225239662185e-02
			r_3 = 6.893883872032158e-02	a_{43} = 1.940500426863389e-01
			r_2 = 3.552631979151575e-01	a_{54} = 3.552631979151575e-01
	0.25	11.0001	r_5 = 2.259782551081724e-04	a_{21} = 3.468110267353592e-02
			r_4 = 6.515890144421776e-03	a_{32} = 9.340016296214622e-02
			r_3 = 6.976315605640404e-02	a_{43} = 1.958382737990192e-01
			r_2 = 3.562284057303278e-01	a_{54} = 3.562284057303278e-01
	0.50	15.5997	r_5 = 6.187625698436672e-05	a_{21} = 2.454451425521160e-02
			r_4 = 2.520981117857257e-03	a_{32} = 6.587052882448075e-02
			r_3 = 3.827176072283689e-02	a_{43} = 1.391840339679659e-01
			r_2 = 2.749723487080808e-01	a_{54} = 2.749723487080808e-01

Notes on the mitogen-activated protein kinase (MAPK) cascade simulation results

The simulation results presented for the mitogen-activated protein kinase (MAPK) cascade were obtained using the following model proposed by Huang and Ferrell:

$$E_1 + KKK \underset{k_2^1}{\overset{k_1^1}{\rightleftarrows}} E_1KKK, \qquad E_1KKK \xrightarrow{k_3^1} E_1 + KKK_P,$$

$$E_2 + KKK_P \underset{k_2^2}{\overset{k_1^2}{\rightleftarrows}} E_2KKK_P, \qquad E_2KKK_P \xrightarrow{k_3^2} E_2 + KKK,$$

$$KKK_P + KK \underset{k_2^3}{\overset{k_1^3}{\rightleftarrows}} KKK_PKK, \qquad KKK_PKK \xrightarrow{k_3^3} KKK_P + KK_P,$$

$$KKPase + KK_P \underset{k_2^4}{\overset{k_1^4}{\rightleftarrows}} KKPaseKK_P, \qquad KKPaseKK_P \xrightarrow{k_3^4} KKPase + KK,$$

$$KKK_P + KK_P \underset{k_2^5}{\overset{k_1^5}{\rightleftarrows}} KKK_PKK_P, \qquad KKK_PKK_P \xrightarrow{k_3^5} KKK_P + KK_{PP},$$

$$KKPase + KK_{PP} \underset{k_2^6}{\overset{k_1^6}{\rightleftarrows}} KKPaseKK_{PP}, \qquad KKPaseKK_{PP} \xrightarrow{k_3^6} KKPase + KK_P,$$

$$K + KK_{PP} \underset{k_2^7}{\overset{k_1^7}{\rightleftarrows}} KK_{PP}K, \qquad KK_{PP}K \xrightarrow{k_3^7} KK_{PP} + K_P,$$

$$KPase + K_P \underset{k_2^8}{\overset{k_1^8}{\rightleftarrows}} KPaseK_P, \qquad KPaseK_P \xrightarrow{k_3^8} KPase + K,$$

$$KK_{PP} + K_P \underset{k_2^9}{\overset{k_1^9}{\rightleftarrows}} KK_{PP}K_P, \qquad KK_{PP}K_P \xrightarrow{k_3^9} KK_{PP} + K_{PP},$$

$$KPase + K_{PP} \underset{k_2^{10}}{\overset{k_1^{10}}{\rightleftarrows}} KPaseK_{PP}, \qquad KPaseK_{PP} \xrightarrow{k_3^{10}} KPase + K_P$$

The parameters used for the simulation are $k_1^i = k_2^i = 150$, $k_3^i = 0.000415076, i = 1, \ldots, 10$. This set of parameters renders the model stiff and with species amounts ranging from one or to molecules up to $3 \cdot 10^5$, thus forcing the τ-leap method to use a very small time step.

The initial conditions for the simulations are set to $E_1 = 72$, $E_2 = 722$, $KKK = 7227$, $KKK_P = 0$, $KK = 2891040$, $KK_P = 0$, $KK_{PP} = 0$, $K = 2891040$, $K_P = 0$, $K_{PP} = 0$, $KPase = 289104$, $KKPase = 722$, $E_1KKK = 0$, $E_2KKK_P = 0$, $KKK_PKK = 0$, $KKK_PKK_P = 0$, $KK_{PP}K = 0$, $KK_{PP}K_P = 0$, $KKPaseKK_{PP} = 0$, $KKPaseKK_P = 0$, $KPaseK_{PP} = 0$ and $KPaseK_P = 0$. We have run the simulations for time form 0 to 200, where the model settles into a stationary state after an initial transient change.

In order to compare the accuracy of the different methods we have run them using different time steps and we have computed the relative variance of one of the species, KK_P, at the stationary state (Figure 1).

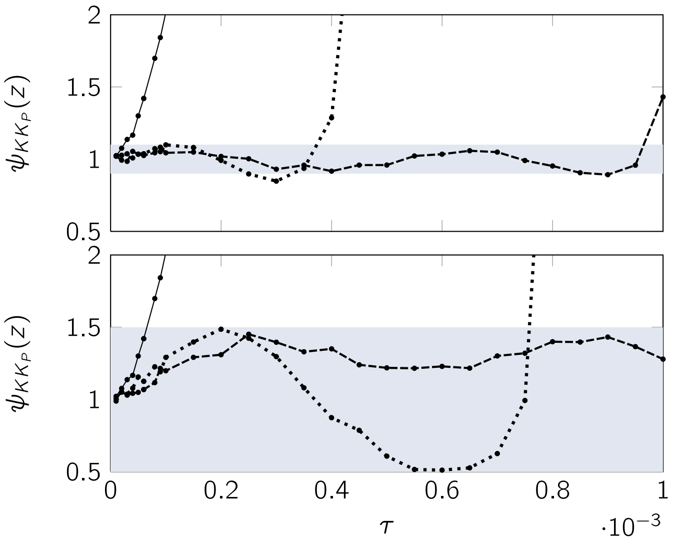

Figure 1: Relative variance of KK_P in the MAPK cascade model for the Poisson τ-leap (solid line) and the RK τ-leap methods with 3 stages (dotted line) and 5 stages (dashed line). **(a)** RK with $\epsilon = 0.1$. **(b)** RK with $\epsilon = 0.5$.
